# Intranasal Oxytocin for Stimulant Use Disorder Among Male Veterans Enrolled in an Opioid Treatment Program: A Randomized Controlled Trial

**DOI:** 10.3389/fpsyt.2021.804997

**Published:** 2022-01-17

**Authors:** Christopher S. Stauffer, Salem Samson, Alex Hickok, William F. Hoffman, Steven L. Batki

**Affiliations:** ^1^Department of Mental Health, VA Portland Health Care System, Portland, OR, United States; ^2^Social Neuroscience and Psychotherapy Lab, Department of Psychiatry, Oregon Health and Science University, Portland, OR, United States; ^3^Department of Psychiatry and Behavioral Sciences, San Francisco School of Medicine and San Francisco VA Health Care System, University of California, San Francisco, San Francisco, CA, United States; ^4^School of Nursing, Massachusetts General Hospital (MGH) Institute of Health Professions, Boston, MA, United States

**Keywords:** oxytocin, amphetamine-related disorders, opioid-related disorders, opiate substitution treatment, treatment adherence and compliance, stimulant, methadone, veterans

## Abstract

The increasing prevalence of illicit stimulant use among those in opioid treatment programs poses a significant risk to public health, stimulant users have the lowest rate of retention and poorest outcomes among those in addiction treatment, and current treatment options are limited. Oxytocin administration has shown promise in reducing addiction-related behavior and enhancing salience to social cues. We conducted a randomized, double-blind, placebo-controlled clinical trial of intranasal oxytocin administered twice daily for 6 weeks to male Veterans with stimulant use disorder who were also receiving opioid agonist therapy and counseling (*n* = 42). There was no significant effect of oxytocin on stimulant use, stimulant craving, or therapeutic alliance over 6 weeks. However, participants receiving oxytocin (vs. placebo) attended significantly more daily opioid agonist therapy dispensing visits. This replicated previous work suggesting that oxytocin may enhance treatment engagement among individuals with stimulant and opioid use disorders, which would address a significant barrier to effective care.

## Introduction

Stimulant use among individuals seeking treatment for opioid use disorder (OUD) has drastically increased over the last decade (1). Co-use of cocaine and/or methamphetamine with opioids elevates the risk of fatal overdose and is associated with poorer medical, mental health, and substance use disorder (SUD) treatment outcomes (2). While there are effective medications to treat OUD, including methadone and buprenorphine (3, 4), there are still no Food and Drug Administration (FDA)-approved medications for stimulant use disorder. Furthermore, clinical trials investigating new treatments for stimulant use disorder typically exclude individuals with OUD (5, 6). A recent systematic review of available clinical trials targeting stimulant use among people with co-occurring OUD reported 21 medications studied for cocaine use and only one medication for methamphetamine use (1); none of the medications studied demonstrated clear benefits.

Epidemiological research suggests that more than a third of all U.S. military Veterans meet criteria for any SUD, excluding tobacco use disorder, over their lifetime (7). Furthermore, lifetime prevalence of SUDs is higher among Veterans vs. non-veterans, and Veterans with a SUD diagnosis reported the lowest levels of functioning across multiple domains—including physical, emotional, and social functioning—compared to Veterans without SUDs or non-veterans with or without SUDs (7). Therefore, Veterans are particularly in need of innovative treatment options for SUDs.

Oxytocin is a hypothalamic peptide hormone which acts both peripherally and centrally and plays a prominent role in social attachment. A body of research suggests that a well-functioning endogenous oxytocinergic system is protective against the development of SUDs, and, conversely, that chronic substance use leads to dysregulation within the oxytocinergic system (8). Animal researchers began exploring oxytocin's anti-addiction effects over 40 years ago (9). In animal models of addiction—including stimulants and opioids—administration of exogenous oxytocin has demonstrated broad benefits, including: prevention and mitigation of drug self-administration, reduced stress- and drug-primed reinstatement of drug self-administration, and reduced signs of withdrawal and tolerance (10–12). Interestingly, laboratory animals housed socially together, vs. isolated in individual cages, respond more robustly to oxytocin administration on substance-related outcome measures (13), supporting the theory that social context can moderate the effects of oxytocin (14). Veterans are more likely to be socially avoidant compared to the general population, thus treatment interventions that promote social attachment may be particularly pertinent to Veterans (15).

More recently, human subjects research has begun to explore the effects of intranasal oxytocin on addiction-related outcomes for various substances of misuse (16, 17). As far as reduction in substance craving and use, results from these clinical trials have been largely underwhelming. Most of these trials administered only a single dose of oxytocin and/or did not pair oxytocin with a psychosocial treatment intervention. Exceptions to these limited trial designs include early phase trials of: (a) intranasal oxytocin vs. placebo administered twice daily for 2 weeks to individuals with cocaine use disorder concurrently enrolled in an opioid treatment program (OTP) for OUD (18) and (b) intranasal oxytocin vs. placebo paired with 6 weekly sessions of motivational interviewing group therapy for methamphetamine use disorder (19). While the first study showed a small effect of oxytocin vs. placebo on self-reported reduction in cocaine use, there was no significant effect of oxytocin on urine levels of cocaine metabolite (18); the second study showed no effect of oxytocin on methamphetamine use (19). Neither study detected a significant effect of oxytocin on stimulant craving or urge to use. Given promising animal data and early mixed data among human subjects, more research is needed to better understand the effects of intranasal oxytocin on SUDs.

Interestingly, a previously unpublished exploratory analysis of Stauffer et al.'s (18) pilot study of oxytocin for co-occurring cocaine use disorder and OUD found that male participants (*n* = 12) demonstrated significantly fewer clinic absences over three weeks when receiving oxytocin vs. placebo (Cohen's *d* = 1.44; *p* = 0.05). Another interesting finding from this study was that participants receiving oxytocin, but not those receiving placebo, demonstrated a significant association between self-reported cocaine use and quantitative urine levels of cocaine metabolite—suggesting that oxytocin may enhance honesty with providers. These exploratory findings infer that oxytocin improves engagement with clinical treatment, specifically treatment attendance and therapeutic alliance, despite no promising short-term effects on stimulant use and craving. Therapeutic alliance refers to the quality of the bond between a patient and therapist, measured through agreement on goals, ways to attain goals, and trust (20).

Subsequently, Stauffer et al. (19) found a significant effect of oxytocin on attendance at group therapy sessions for methamphetamine use disorder (OR 3.26, 95% CI [1.27–8.41], *p* = 0.014; *n* = 48, all male-identified). This trial also found positive effects of oxytocin on aspects of group cohesion (19) and physiological synchrony (21); although oxytocin had no significant effect on methamphetamine use or craving after 6 weeks of treatment. Of note, endogenous oxytocin has been nominated as a possible biomarker for therapeutic alliance (22); and—regardless of the therapeutic modality—the strength of the therapeutic alliance consistently predicts addiction treatment engagement and retention as well as long-term relapse (7, 23). Thus, it is important that we gain a better understanding of the relationship between oxytocin and therapeutic alliance, particularly among individuals with SUDs in controlled therapeutic environments (24, 25). Lastly, some research has suggested that adverse childhood experiences can moderate the effects of intranasal oxytocin among individuals with SUDs (26, 27).

The current study investigates the effects of intranasal oxytocin vs. placebo administered to Veterans with stimulant use disorder in the context of receiving care at an OTP for OUD. The primary clinical outcome is change in stimulant use, using both self-report and urine drug test. Secondary outcomes include: (a) stimulant craving, (b) therapeutic alliance with OTP counselor, and (c) OTP clinic attendance. We hypothesized that administration of oxytocin vs. placebo would result in reduced stimulant use and craving and improved therapeutic alliance and clinic engagement.

## Materials and Methods

### Trial Design

We conducted a randomized, double-blind, placebo-controlled, clinical trial (NCT03016598) of intranasal oxytocin administered twice daily for 6 weeks. The study was approved by the University of California, San Francisco Institutional Review Board (IRB) and was conducted according to Good Clinical Practices.

### Participants and Recruitment

#### Eligibility Criteria

Participants included in the study were (a) Veterans, (b) ≥18 years old, (c) enrolled in an OTP and on a stable dose of opioid agonist therapy (methadone or buprenorphine) for at least 2 weeks, (d) with severe stimulant use disorder according to the *Diagnostic and Statistical Manual of Mental Disorders*, Fifth Edition (*DSM-*5) criteria, and (e) with a documented urine toxicology test positive for stimulant use (cocaine and/or methamphetamine) in the past year.

We excluded participants who had (a) active suicidal or homicidal ideation, (b) conditions preventing nasal spray administration (e.g., nasal obstruction, frequent nosebleeds), or (c) known allergic reaction or sensitivity to the preservatives in the nasal spray.

#### Recruitment and Screening

Participants were recruited between January 2018 and February 2020 from two OTPs in the San Francisco Veterans Affairs (VA) Health Care System, the San Francisco VA Medical Center and the Oakland Behavioral Health Clinic. Potential participants were recruited through referrals from OTP counselors and flyers advertising the study posted within the OTP clinics.

To determine preliminary eligibility, staff conducted brief, structured, in-person interviews with interested participants. Preliminarily eligible Veterans were then invited to complete a full screening assessment to determine eligibility for study participation. Study staff obtained informed consent prior to conducting any study procedures. A trained clinical interviewer with at least Masters' level training in clinical psychology conducted pertinent diagnostic interviews from the Mini International Neuropsychiatric Interview (MINI) 7.0.0 (28) and a structured interview to determine lifetime and 30-day frequency of substance use (29). A study physician performed an examination of the nasal parenchyma. Participants also completed a demographics interview and the Adverse Childhood Experience (ACE) questionnaire—for which higher scores indicate a greater number of adverse childhood experiences, such as emotional, physical, and sexual abuse, and emotional and physical neglect (30).

Participants were compensated a total of $50 for completing the screening visit and up to an additional $300 for full participation in the study. Compensation was $50 per week, $30 of which they received at each of 6 weekly visits and $20 of which was added to a completion bonus disbursed at the sixth and final visit.

#### Randomization and Blinding

Enrolled participants were randomly allocated by the research pharmacist to receive either oxytocin or placebo (1:1) throughout the study intervention period. Participants and study staff were kept blinded to study condition until the final participant completed study termination.

### Procedures

#### Study Drug

Oxytocin is a large hydrophilic molecule that does not cross the blood-brain-barrier in appreciable amounts when administered peripherally. However, intranasal administration is thought to reach the brain via various pathways, acutely resulting in elevated oxytocin levels in the cerebrospinal fluid and measurable behavioral effects in the laboratory for up to a few hours (31). Participants received oxytocin 40 International Units (IU) or placebo intranasally twice daily for 6 weeks. Oxytocin was purchased from Valor Compounding Pharmacy (Berkeley, CA, USA). Oxytocin concentration was 40 IU/0.5 mL. Study drug was administered in clinic every morning using a mucosal nasal atomizer (MAD300; Teleflex technologies, Mooresville, NC). In the evening—approximately 12 h after the morning dose—as well as every 12 h on days the clinic was closed (e.g., Sunday, holidays), participants self-administered study drug using a bottle with a metered-dose nasal spray pump (Aptar Classic Technology, Crystal Lake, IL). Participants were trained in proper self-administration by study staff. To monitor adherence, nasal spray bottles were weighed prior to and after weekly participant use and a timeline follow-back (TLFB) procedure was conducted for self-administered evening dosing over the prior week. Participants were incentivized to bring their bottle back for weighing, regardless of how many doses they'd self-administered, by the loss of $10 from their weekly compensation if they forgot.

#### Assessments

Following enrollment, participants attended a baseline and 6 additional weekly assessments. During weekly assessments, study staff asked about stimulant use and cravings over the prior week and collected a urine sample to evaluate for stimulant use. At the baseline and final assessments, each participant and their respective OTP counselor completed an assessment of therapeutic alliance. See Table 1 for timing of measurements.

**Table 1:** Timing of measurements.

	**Screening**	**1**	**2**	**3**	**4**	**5**	**6**	**7**
Oxytocin 40 IU vs Placebo intranasally		Twice daily x 6 weeks						
**Screening assessment/baseline characteristics**
Mini international neuropsychiatric interview	X							
Demographics	X							
Adverse childhood experiences (ACE)	X							
Lifetime substance use	X							
30-day substance use	X							
**Outcome measures**
Self-reported stimulant use (timeline Follow-back)			X	X	X	X	X	X
Urine drug test	X	X	X	X	X	X	X	X
Cocaine craving questionnaire-brief (CCQ-Br)	X	X	X	X	X	X	X	X
Working alliance inventory-short revised (WAI-SR)		X						X
WAI-SR-T (therapist)		X						X
Clinic attendance		Daily x 6 weeks						

*IU, International Units. 1, Baseline assessment, 2–7, Weekly assessments*.

### Outcome Measures

#### Primary Clinical Outcome—Stimulant Use

##### Self-Reported Stimulant Use

The Timeline Follow-back is a structured interview conducted by study staff to determine the number of days over the past week, including the day of the interview, that participants used a stimulant (32, 33).

##### Urine Drug Testing

We used a point-of-care, CLIA-waived, 10-panel, Toxicology iCup Dx (Alere Inc., Waltham, MA) to measure stimulant use (cocaine and/or methamphetamine).

#### Secondary Outcome Measures

##### Stimulant Craving

The self-report Stimulant Craving Questionnaire-Brief (STCQ-Br) measures current general stimulant craving (34). Each of the 10 items is scored on a 7-point Likert scale. Adaptation of the STCQ-Br for the current study involved replacing the word “stimulant” in each item with the individual's preferred term for their stimulant of choice, which was collected during screening.

##### Therapeutic Alliance

We used the Working Alliance Inventory-Short Revised (WAI-SR) and the Working Alliance Inventory-Short Revised-Therapist Version (WAI-SR-T) to measure therapeutic alliance between participant and their OTP counselor (20). These 12-item self-report questionnaires use a 7-point Likert scale to rate therapeutic alliance based on three elements: the degree to which both parties agree on the goals of treatment, agreement on the tasks to attain those goals, and the development of trust (35).

##### Clinic Attendance

OTP clinic attendance was measured as the proportion of required daily opioid agonist dosing visits that the participant attended during the 6-week study intervention period. One study site required 6 days per week attendance and the other site required 5 days per week attendance for OTP patients at the initial phase of care. Two participants were higher phase and did not require daily OTP attendance; thus, they were removed from analysis for this outcome.

### Analysis Plan

#### Sample Size Calculation

The initial sample size calculation determined that 25 participants in each group would have 81% power over 6 weeks to detect the small-medium effect size (*d* = 0.31) found in our pilot work for between-group difference in urine toxicology and >99% power to detect the large effect size (*d* = 1.44) we found in our pilot work for between-group difference in OTP attendance.

#### Statistical Methods

All analyses were conducted with R (version 3.6.3) and all confidence intervals (CIs) are reported at the 95% coverage level (36). Covariates were chosen based on bivariate analysis between groups as to reduce confounding by differing demographics.

Stimulant use (measured by TLFB) and self-reported craving (measured by STCQ-Br) were modeled using a linear mixed model with random intercepts for patients to account for repeated weekly measurements over the study period. The primary predictor of interest was the interaction between study drug (oxytocin vs. placebo) and week of the trial (1–6) to examine the effectiveness of oxytocin over the course of the trial. Covariates included age (continuous), race (white, black, other), and smoking status (smoker/non-smoker). Race was simplified due to small numbers of patients in non-White or non-Black categories. For the self-reported craving outcome, the model was adjusted for craving at the baseline visit. Complete cases were used in analysis, resulting in *n* = 40 for TLFB and *n* = 39 for STCQ-Br. One patient was missing a baseline STCQ-Br score and thus excluded from this analysis.

Weekly urine toxicology results (either positive or negative for stimulants) and weekly clinic attendance (proportion of OTP dosing sessions attended) was modeled using a generalized estimating equation (GEE) with a logit link for a binomial distribution. An autoregressive (level one) covariance structure was used to account for repeated measures. The primary predictor of interest was the interaction between study drug use and week of the trial. Covariates included age (continuous), race (white, black, other), and smoking status (smoker/non-smoker). Complete cases were used in analysis, resulting in *n* = 40 patients for these outcomes.

Change in the WAI-SR and WAI-SR-T was measured by taking the difference in the scores at week seven minus the scores at baseline. A positive change indicates an increase in the strength of the therapeutic alliance. This change was modeled with a linear fixed effects model, with the study drug as the primary predictor of interest. Covariates included age, race, smoking status, and the ACE sum score. In our exploratory analysis and model building process, we did not find any significant relationship between ACE and stimulant-related outcomes, nor did it appear to have a noticeable confounding effect on other covariates. As such, ACE was only used in conjunction with therapeutic alliance in order to present more parsimonious models. Complete cases were used in analysis, resulting in *n* = 38 patients for the WAI-SR outcome, and *n* = 37 patients for the WAI-SR-T outcome. Missing observations were due to missing surveys at week seven.

## Results

### Participants

**Figure 1:**
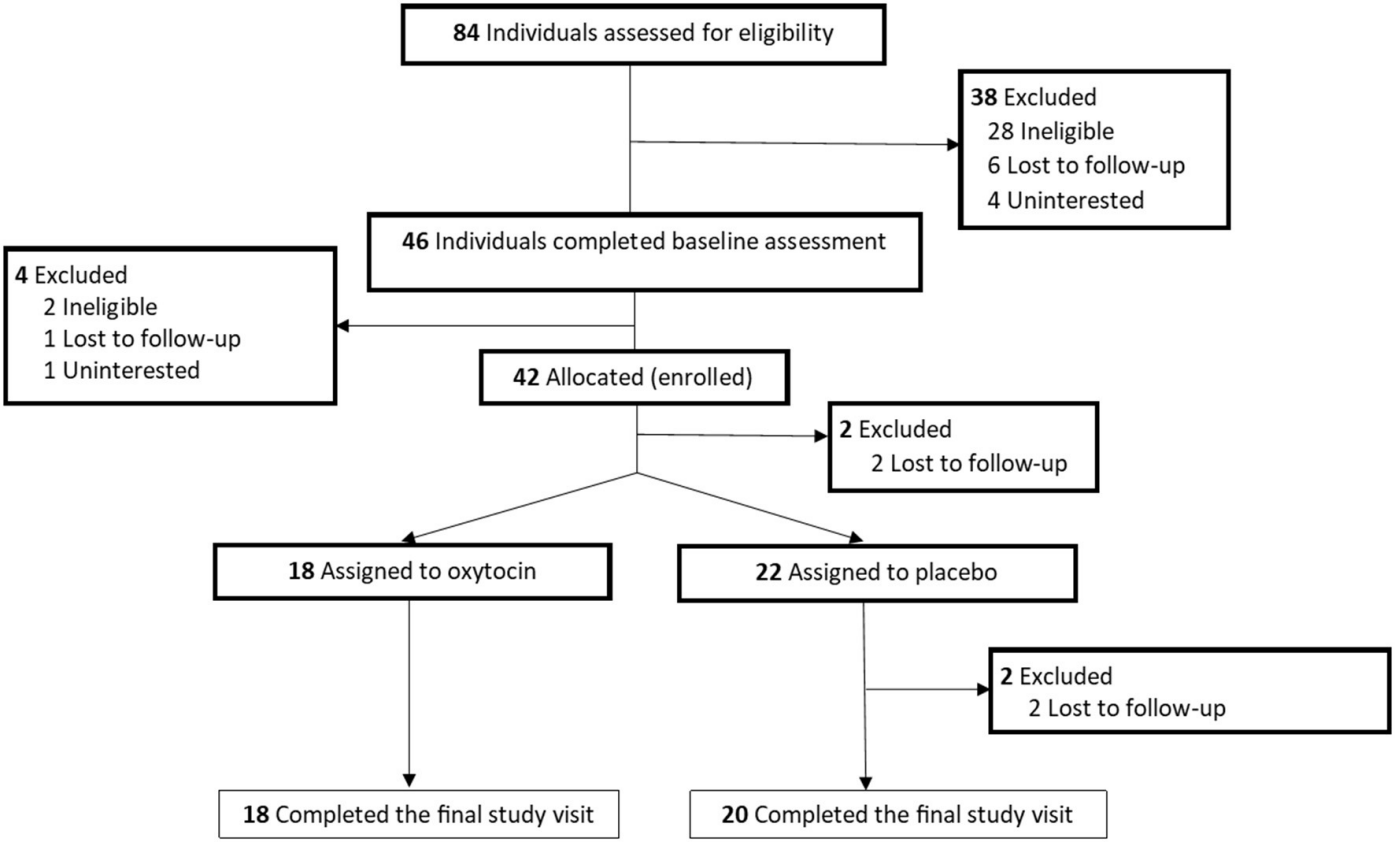
Participant flow diagram.

See Figure 1 for participant flow diagram (37). Of note, we did not meet our initial goal of 50 participants. We noted a lack of eligible participants at our primary site and gained regulatory approval to recruit from an additional site. Ultimately, we enrolled 42 participants within the grant period. See Table 2 for demographics and baseline characteristics. While females were not excluded from participating in the study, no female participants were recruited. Generally, participants receiving placebo were older, included a higher percentage of black participants, a higher percentage of cocaine users (vs. methamphetamine users), a lower percentage of smokers, and included no participants who were without housing in the previous year (compared to *n* = 5 from the oxytocin treatment arm).

**Table 2:** Demographics and baseline characteristics.

		**Oxytocin**	**Placebo**	**Overall**
		**(n = 18)**	**(n = 22)**	**(n = 40)**
**Age**; Mean (SD)		53.9 (11.4)	63.1 (7.3)	59 (10.3)
**Gender Identity**			
	Male; *n* (%)	18 (100%)	22 (100%)	40 (100%)
**Kinsey score**^a^; Mean (SD)		0.33 (1.4)	0.18 (0.7)	0.25 (1.1)
**Race;** ***n*** **(%)**			
	African American/Black	7 (38.9%)	17 (77.3%)	24 (60.00%)
	Multiracial	4 (22.2%)	1 (4.5%)	5 (12.5%)
	Native American/Pacific Islander	0 (0.0%)	1 (4.5%)	1 (2.5%)
	White	7 (38.9%)	3 (13.6%)	10 (25.0%)
**Ethnicity**	Hispanic/Latino; *n* (%)	2 (11.1%)	2 (9.1%)	4 (10.0%)
**Education;** ***n*** **(%)**			
	≤ High school graduate	2 (11.1%)	4 (18.2%)	6 (15.0%)
	High school grad	5 (27.8%)	8 (36.4%)	13 (32.5%)
	Some college/Trade	10 (55.6%)	9 (40.9%)	19 (47.5%)
	Bachelor's Degree	1 (5.6%)	1 (4.6%)	2 (5.0%)
**Annual income**; *n* (%)	≤ $11,880^b^	5 (27.8%)	4 (18.2%)	9 (22.5%)
**Employed**; *n* (%)		2 (11.1%)	2 (18.2%)	4 (10.0%)
**Disability**; *n* (%)		14 (77.8%)	16 (72.7%)	30 (75.0%)
**Housing**	Houseless past year; *n* (%)	5 (27.8%)	0 (0.0%)	5 (12.5%)
**Relationship status**	Primary relationship^c^; *n* (%)	5 (27.8%)	3 (13.6%)	8 (20.0%)
**Smoking status**	Smoker; *n* (%)	17 (94.4%)	15 (68.2%)	32 (80.0%)
**Opioid agonist therapy**	Methadone (vs. buprenorphine); *n* (%)	16 (88.9%)	17 (77.3%)	33 (82.5%)
**Stimulant of choice**	Cocaine (vs. methamphetamine); *n* (%)	12 (66.7%)	19 (86.4%)	31 (77.5%)
**Years used** **≥3 times per week/Age**; Mean (SD)	Cocaine	0.25 (0.2)	0.23 (0.2)	0.24 (0.2)
	Methamphetamine	0.11 (0.2)	0.05 (0.2)	0.10 (0.2)
**Proportion of days used in past 30 days**; Mean (SD)	Cocaine	0.14 (0.3)	0.25 (0.4)	0.20 (0.3)
	Methamphetamine	0.12 (0.2)	0.08 (0.2)	0.07 (0.2)
**Stimulant craving**; Mean (SD)	[range: 1–7]	2.14 (1.3)	1.92 (0.9)	1.99 (1.0)
**Therapeutic alliance**; Mean (SD) [range: 1–5]
	Participant	3.60 (0.9)	3.82 (0.8)	3.72 (0.8)
	Therapist	4.09 (0.7)	3.75 (0.7)	3.91 (0.7)
**Adverse childhood experiences**; Mean (SD)	[range: 0–10]	3.5 (2.5)	4.55 (2.7)	4.08 (2.6)

a *scale from “0, exclusive heterosexuality” to “6, exclusive homosexuality”*.

b *2016 United States Department of Health and Human Services poverty guideline*.

c *Someone with whom you are currently in love or feel a commitment to. SD, standard deviation*.

### Intervention Adherence

Adherence rates for morning clinic-administered and evening self-administered study drug dosing is as follows: 92.0 and 84.1% for oxytocin, respectively, and 85.4 and 90.2% for placebo. The mean (SD) differences in bottle weight (mg) following each week of use were: Oxytocin 3.1 (1.7) and Placebo 3.2 (1.6).

### Outcomes

#### Primary Outcome—Stimulant Use

##### Self-Reported Stimulant Use

**Figure 2:**
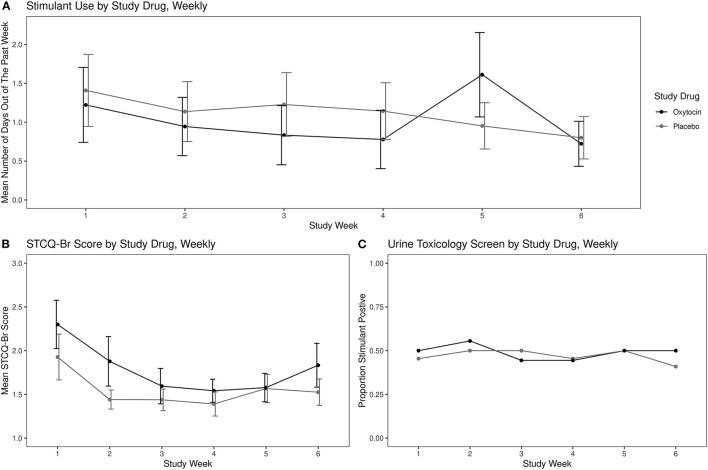
Stimulant use and craving by study drug: **(A)** mean self-reported stimulant use using the Timeline Follow-back, **(B)** mean self-reported stimulant craving using the Stimulant Craving Questionnaire-Brief (STCQ-Br), and **(C)** proportion stimulant-positive urine toxicology. Error bars, Standard Error of the Mean.

For the overall sample, there was a significant reduction in stimulant use as the trial progressed by 0.10 days per week (CI: −0.19 to −0.02; *p* = 0.02), but there was no significant effect for the study drug by week interaction (estimate: 0.08; CI: −0.04 to 0.21; *p* = 0.19). None of the model covariates were significantly associated with the outcome. See Figure 2A.

##### Urine Toxicology

There was no significant difference in proportion of positive weekly urine toxicology screens over the study period between the study drug groups (OR: 0.96; CI: 0.88–1.04; *p* = 0.32). None of the model covariates were significantly associated with the outcome. See Figure 2C.

#### Secondary Outcomes

##### Stimulant Craving

Overall, there was a significant decrease in reported craving by week over the course of the study period of 0.07 points per week (CI: −0.13 to −0.01; *p* = 0.02), but there was no significant effect for the study drug by week interaction (estimate: −0.02; CI: −0.11 to 0.07; *p* = 0.64). See Figure 2B.

##### Therapeutic Alliance

**Figure 3:**
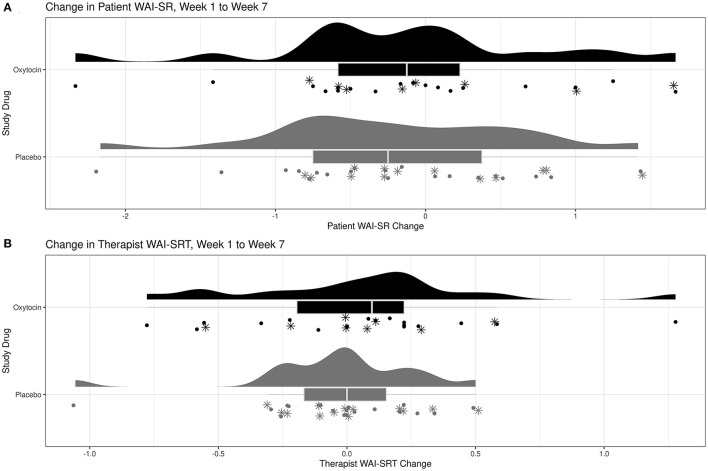
Change in therapeutic alliance after six weeks of oxytocin vs. placebo using the Working Alliance Inventory-Short Revised (WAI-SR): **(A)** WAI-SR, patient version and **(B)** WAI-SR-T, therapist version. *Participants with ACE ≥4.

Overall, there was not a significant relationship with study drug and change in patient WAI-SR score (estimate: 0.06; CI: −0.63 to 0.75; *p* = 0.86). See Figure 3A. Interestingly, those patients with higher ACE sum scores did see a significant increase in average WAI-SR score regardless of study drug (estimate: 0.14; CI: 0.02–0.25; *p* = 0.023). Interaction between study drug and ACE was tested but not significant and not reported for the final model. There was also no significant effect of study drug on change in therapist WAI-SR-T score (estimate: −0.02; CI: −0.38 to 0.34; *p* = 0.91). See Figure 3B. In contrast to the patient score change, the change in therapist rating did not have any significant relationship with baseline ACE or any other covariates.

##### Clinic Attendance

**Figure 4:**
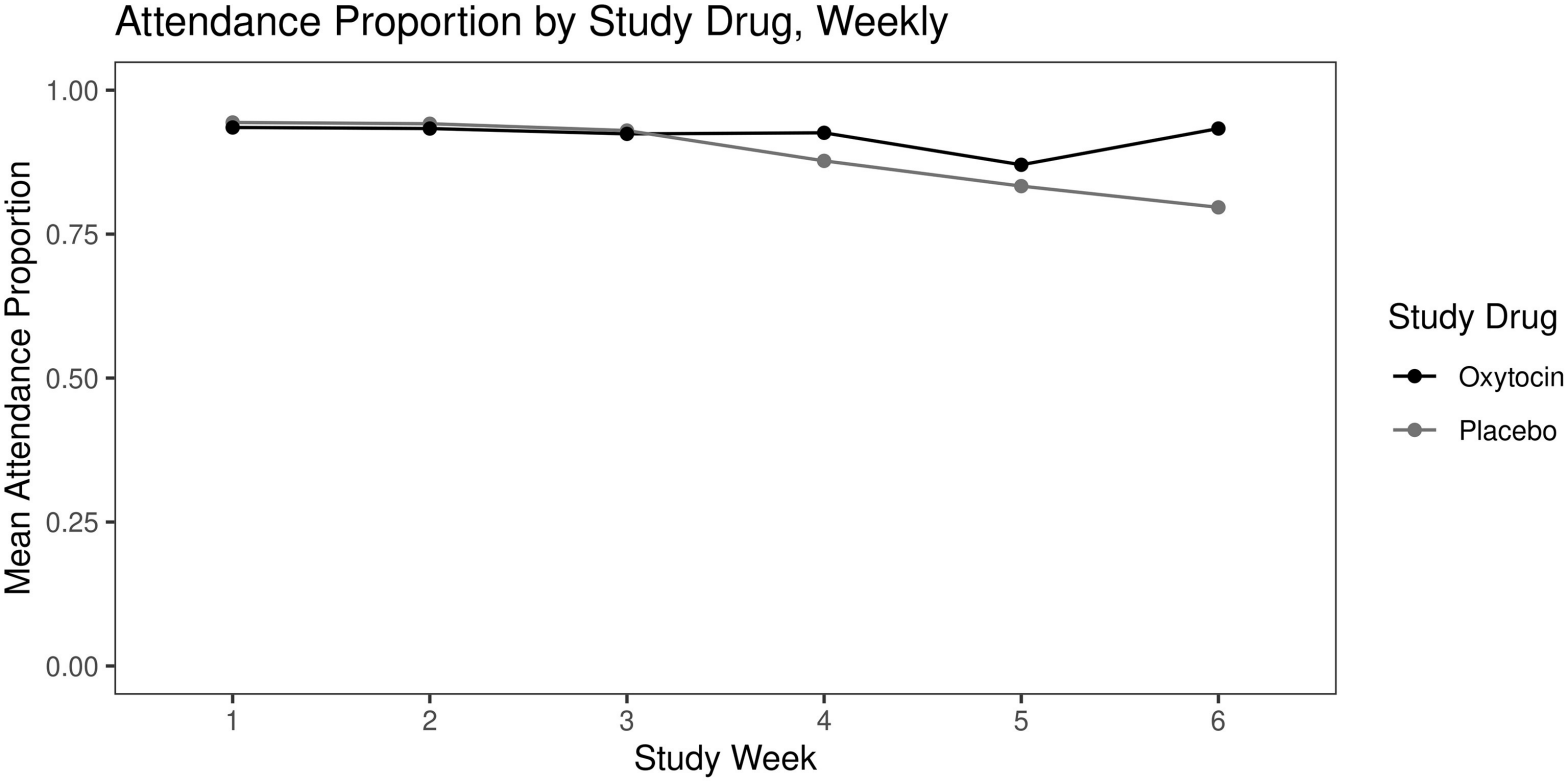
Mean opioid treatment program clinic attendance (proportion of weekly scheduled visits attended) over 6 weeks by study drug.

Overall, there was a significant decrease in proportion of clinic attendance by week (OR: 0.70; CI: 0.53–0.94; *p* = 0.015). There was a significant interaction of study drug and week, in that those patients receiving oxytocin had higher attendance rates compared to those who received placebo as the study progressed (OR: 1.39; CI: 1.04–1.86; *p* = 0.03). See Figure 4.

## Discussion

Contrary to our hypothesis, twice daily dosing of oxytocin vs. placebo over 6 weeks did not affect stimulant use as evidenced by self-report and urine drug test among Veterans with stimulant use disorder within an OTP. There was also no effect of oxytocin on our measurements of stimulant craving or therapeutic alliance. Regardless of study drug, there was a significant reduction in self-reported stimulant use and craving over the 6 weeks; however, there was no significant change in stimulant-positive urine tests. Overall, having more adverse childhood experiences was significantly associated with improved therapeutic alliance over the course of the study, but there was no interaction with oxytocin. While oxytocin had no noticeable effects on our substance-related outcome measures or therapeutic alliance, participants receiving oxytocin attended significantly more OTP clinic visits compared to participants receiving placebo. This finding replicates earlier work showing that oxytocin administration was associated with fewer absences in addiction treatment settings (18, 19), suggesting that oxytocin may enhance treatment engagement among stimulant users.

While a large body of preclinical evidence has reliably shown that oxytocin administration reduces stimulant use and related behavior, these outcomes have not translated clearly to human clinical trials. In the present study, we expect the placebo effect contributed to the reduction in stimulant use and craving over time within both treatment arms, in addition to related phenomena such as regression to the mean, spontaneous remission, outcome expectancies, and the Hawthorne effect—or changing behavior as a response to attention received through observation and assessment (38). Of note, our sample consists of relatively chronic users (having used stimulants three or more times per week for 10–24% of their lives on average), and our 6-week assessment period was relatively brief. Nonetheless, we recognize the importance of publishing null results in moving the field forward (31).

This is the third clinical trial among individuals with stimulant use disorder to demonstrate a protective effect of oxytocin on dwindling clinic attendance over time among male participants (18, 19). Generally, dropout rates are notably higher among stimulant users compared to other SUDs (39), and 40–62% of Veterans fall out of care before completing a predetermined course of outpatient addiction treatment (40, 41). Furthermore, no significant differences in treatment retention exist between evidence-based, addiction-focused, psychosocial treatments (e.g., motivational interviewing, contingency management, cognitive-behavioral therapy) and standard care (42)—highlighting a lack of options available to address these retention issues. Perhaps obviously, a body of evidence has shown that the effectiveness of addiction treatment is weakened significantly by early dropout (43, 44). For example, community addiction treatment duration of <90 days was associated with significantly less favorable outcomes 1 year later, and single episode treatment duration beyond 90 days had a linear relationship with positive treatment outcomes at 1 year (45). Unfortunately, the current trial did not involve any follow-up assessment beyond our 6-week intervention. In a meta-analysis of medication trials for co-occurring stimulant use disorder and OUD (1), only one intervention—naltrexone implant (46)—demonstrated a positive effect on retention compared to placebo. Most other interventions had no effect on retention; while antidepressants, anticonvulsants, and disulfiram worsened retention compared to placebo (1). Conversely, intranasal oxytocin and naltrexone, a μ-opioid antagonist, may act synergistically to improve retention (46–48), and the combination warrants further investigation. Because retention in addiction treatment has generally been associated with improved long-term treatment outcomes, and there is a scarcity of available interventions to effectively address critically high dropout rates among stimulant users, further research into oxytocin's potential to improve treatment engagement is warranted.

We saw an association between adverse childhood experiences and improved therapeutic alliance over the course of our intervention. While some research has suggested that adverse childhood experiences can moderate the effects of intranasal oxytocin (26, 27), our study did not find such an effect. Nonetheless, the social salience hypothesis of oxytocin posits that, rather than having purely prosocial effects, oxytocin modulates social responsivity based on both external contextual social cues (e.g., competitive vs. cooperative environments) and individual characteristics (e.g., history of interpersonal trauma, gender, sexual orientation) (14). This highlights the potential importance of a model that pairs oxytocin dosing with supportive psychosocial treatment, rather than the typical psychopharmacology model of routine self-administration in uncontrolled social contexts. In the current study, participants' morning doses were administered by friendly staff in a clinic setting; however, the social context of their evening dosing was not controlled. On the other hand, Stauffer et al. (19) paired oxytocin administration solely with motivational interviewing group therapy for methamphetamine use disorder and saw positive effects on attendance and therapeutic alliance within 6 weeks. Flanagan et al. (49) are currently conducting a Phase II clinical trial (*N* = 200) of oxytocin vs. placebo paired with Alcohol Behavioral Couples Therapy (49). We suggest that future oxytocin studies continue to explore the effect of social context on clinical outcomes. If intranasal oxytocin enhances perceptions of social support and boosts treatment engagement in supportive social contexts, this may mitigate addiction severity over time. Future studies may also consider qualitative interviews to capture subjective experiences associated with improvements in attendance.

This study has several limitations, including limitations in its design and being underpowered to detect significant changes in the primary clinical outcome. Generalizability does not extend beyond older, male Veterans with chronic stimulant use receiving care within an OTP. While female participants were not excluded from participating, the VA OTP clinics from which we recruited had very few female patients—none of whom met eligibility criteria for study participation. Despite randomization, participant demographics between experimental groups were not well-matched by age, race, or homelessness in the past year. Past 30-day stimulant use and craving at baseline were relatively low in our sample. Both opioid replacement medication type (buprenorphine or methadone) and stimulant of choice (cocaine, methamphetamine, or both) were considered as covariates but ultimately left out as they did not improve model performance or predictive power and were not significantly related to outcomes. Additionally, with our limited sample size, using these variables as covariates presented estimation issues due to imbalances across treatment groups. Oxytocin has a short half-life (~19 minutes) (50) but primes its own release (51, 52), perhaps contributing to prolonged elevation in oxytocin concentrations and behavioral effects after intranasal oxytocin administration (53). However, evidence also suggests that oxytocin release is inhibited by μ-opioid receptor agonists (54, 55) (as opposed to naltrexone, a μ-opioid receptor antagonist mentioned earlier as having potential synergy with oxytocin). Thus, the effects of intranasal oxytocin may be blunted in people receiving opioid agonist therapy with methadone and buprenorphine. Comparison studies of intranasal oxytocin administered to participants with stimulant use disorder both with and without co-occurring OUD are poised to help further our understanding of any clinically pertinent drug-drug interaction between oxytocin and opioids. Finally, we did not account for concomitant medication use or psychiatric diagnoses beyond our eligibility criteria, and our study design did not include any long-term follow-up assessment.

The increasing prevalence of co-morbid stimulant and opioid use poses a significant risk to public health, and current treatment options are limited. Research suggests an inverse relationship between social support and addiction severity (56–59). Twice daily administration of the social neuropeptide oxytocin for up to 6 weeks in a real-world OTP clinic setting did not seem to affect stimulant use or craving. However, we replicated previous findings in which oxytocin maintained engagement with clinical interventions over time among stimulant users (18, 19). These results suggest a potential practical application for intranasal oxytocin in bridging the gap between addiction and social connection (24, 25), which would address a significant barrier to effective care (i.e., particularly high treatment dropout rates among stimulant users). Oxytocin's effects on addiction treatment attendance warrant further investigation, including clinical trials with larger, more diverse samples and follow-up assessments to measure longer-term effects of oxytocin on treatment dropout, therapeutic alliance, and potential changes in substance craving and use beyond 6 weeks.

## Data Availability Statement

The raw data supporting the conclusions of this article will be made available by the authors, without undue reservation.

## Ethics Statement

The studies involving human participants were reviewed and approved by University of California, San Francisco Institutional Review Board. The patients/participants provided their written informed consent to participate in this study.

## Author Contributions

CS: conceptualization, funding acquisition, protocol design, and trained study staff. SS: recruitment manager, study implementation, data collection, and organization. AH: data analysis. WFH and SB: mentorship. All authors reviewed and edited the final manuscript.

## Funding

This study was funded by the Department of Veterans Affairs, Clinical Science Research and Development, Federal Award Identification Number IK2CX001495.

## Conflict of Interest

The authors declare that the research was conducted in the absence of any commercial or financial relationships that could be construed as a potential conflict of interest.

## Publisher's Note

All claims expressed in this article are solely those of the authors and do not necessarily represent those of their affiliated organizations, or those of the publisher, the editors and the reviewers. Any product that may be evaluated in this article, or claim that may be made by its manufacturer, is not guaranteed or endorsed by the publisher.
